# Significance of abnormal umbilical artery Doppler studies in normally grown fetuses

**DOI:** 10.1186/s40748-020-0115-7

**Published:** 2020-02-20

**Authors:** Nebras Abu Al Hamayel, Haitham Baghlaf, Karin Blakemore, Jude P. Crino, Irina Burd

**Affiliations:** Department of Gynecology and Obstetrics, Johns Hopkins University, School of Medicine, 600 N Wolfe St, Phipps 228, Baltimore, MD 21287 USA

**Keywords:** Doppler, Intrauterine growth restriction, Small for gestational age, Umbilical artery Doppler

## Abstract

**Objective:**

To determine whether there is a relationship between abnormal umbilical artery Doppler studies (UADS) and small for gestational age (SGA) birth weight and other adverse perinatal outcomes in fetuses that appear normally grown by ultrasound.

**Methods:**

This was a retrospective study of all women who had UADS performed at or after 26 weeks of gestation at our institution between January 2005 and December 2012. Women were excluded if they had a fetal demise, a fetus with growth restriction, a fetus with congenital anomaly, or a multiple gestation. Women with missing delivery outcomes were excluded. The primary outcome was birth weight below the 10th percentile.

**Results:**

There were 2744 women included in the study. Of those, 98 (3.6%) had an abnormal UADS, and 379 (13.8%) had an SGA neonate. Of the 2646 women who had a normal UADS, 353 (13.3%) women had an SGA neonate. Twenty-six (26.5%) of the 98 women who had an abnormal UADS had an SGA neonate. After adjusting for potential confounders, the adjusted odds ratio for an SGA neonate with an abnormal UADS was 2.2 (95% CI, 1.38–3.58; *p* <  0.05). In examining other adverse perinatal outcomes, neonatal intensive care unit (NICU) admission and low 5-min Apgar scores were 12.4 and 2.3%, respectively. The adjusted odds ratio for NICU admission was 1.84 (95% CI, 1.06–3.21; *p* <  0.05). Abnormal UADS was not associated with low Apgar scores (aOR 1.39: 95% CI 0.47–4.07; *p* > 0.05).

**Conclusions:**

Our data suggest that abnormal UADS in fetuses that appear normally grown by ultrasound are associated with SGA neonates and NICU admission.

## Introduction

Small for gestational age (SGA) birth weight affects 11% of neonates born in the United States, with African Americans being the highest prevalence group at 17% [1]. SGA is an adverse perinatal outcome that contributes to neonatal mortality and morbidity such as polycythemia, hyperbilirubinemia, hypoglycemia, hypothermia, apnea, asphyxia, seizures, and sepsis [2–4]. Additionally, SGA infants have a higher risk of developing chronic diseases later in life, such as diabetes mellitus type 2, hypertension, cardiovascular diseases, and intellectual disability [5–9].

Ultrasound has been used for several decades to estimate fetal weight and to diagnose intrauterine growth restriction (IUGR). Umbilical artery Doppler studies (UADS), a noninvasive measure of the fetal hemodynamic state [10], are beneficial for monitoring growth restricted fetuses in order to predict their associated perinatal outcomes and to further manage them [11–13]. However, the significance of abnormal UADS in a normally grown fetus is not clear. The Society for Maternal-Fetal Medicine, under the *“Choosing Wisely”* campaign, recommends against screening for fetal growth restriction using Doppler studies. Despite such recommendations, physicians and ultrasonographers are sometimes left with abnormal results in normally grown fetuses when performing a Doppler study. Studies that examine the associations between abnormal UADS in normally grown fetuses and adverse perinatal outcomes are limited [14–17].

Our objectives were 1) to determine whether there is a relationship between abnormal UADS in apparently normally grown fetuses and subsequent diagnosis of SGA at birth, and 2) to investigate whether there is an association of abnormal UADS and adverse perinatal outcomes [neonatal intensive care unit (NICU) admission and low Apgar scores] among neonates who were estimated to be normally grown in utero.

## Methods

### Population

We conducted a retrospective cohort study in a single tertiary referral center between January 1st, 2005 and December 31st, 2012. The Johns Hopkins Medicine Institutional Review Board approved the study. We searched our ultrasound database for all women who had an UADS done at 26 weeks of gestation or later. Data and pregnancy outcomes were extracted using maternal and neonatal electronic medical records. Women with normally grown fetuses, who had an UADS performed at or after 26 weeks gestation, were included in the study. We excluded pregnancies that were complicated by multiple gestation, IUGR, congenital anomalies, or intrauterine fetal demise (IUFD). We excluded women with missing delivery outcomes or missing study variables that were included in our final analysis. IUGR was defined as estimated fetal weight less than the 10th percentile based on standardized national growth curves [18].

### Study variables

Our UADS included measurements of systolic-to-diastolic ratio (S/D), pulsatility index (PI), and resistance index (RI). UADS results were classified either 1) normal UADS, where each of S/D, PI, and RI were below the 95th percentile specific to each gestational age, or 2) abnormal UADS, where either S/D, PI, and/or RI were above the 95th percentile specific to each gestational age. Absent and reversed end-diastolic flows were recorded. We compared women who had an “abnormal UADS” to those with a “normal UADS.” When a woman had more than one UADS done, the last one before delivery was included.

Our primary outcome was SGA, defined as birth weight less than the 10th percentile based on national gender-based nomograms [19]. Secondary outcomes were NICU admission and low Apgar scores, defined as an Apgar score less than seven at 5 min after birth. All outcomes were dichotomized. The following variables were considered potential confounding factors and were included in the final analysis: maternal age at delivery, racial-ethnic background, parity, smoking status, fetal gender, and pre-pregnancy body mass index (BMI). BMI was categorized according to standard BMI ranges for adults.

Data were collected on hypertension status, gestational age at delivery, diabetes mellitus status, placenta previa, low-lying placenta, premature rupture of membranes, and mode of delivery. Gestational age was calculated based on the last menstrual period (LMP) unless the LMP was unknown or differed by more than 7 days from a first trimester ultrasound or more than 14 days from a second trimester ultrasound, in which case sonographic gestational age was used.

### Statistical analysis

We performed univariate analysis to compare maternal and obstetric characteristics according to UADS. For comparing groups, we used the two-sample *t*-test or Mann Whitney *U*-test for continuous variables and the chi square test for categorical variables. We used simple and multiple logistic regression analyses to examine the associations. Results were reported using odds ratios (ORs) with 95% confidence intervals (CIs). Potential confounding variables presented in our final model were chosen based on biologic plausibility and different statistical selection procedures. We tested for collinearity between the variables using variance inflation factors and Spearman’s correlation rank coefficients.

The *p* values for hypotheses testing and group comparisons were two-sided and the significance level was set at *p* <  0.05. Statistical analysis was performed using Stata 13.1 (StataCorp. 2013. Stata Statistical Software: Release 13. College Station, Texas).

## Results

During the study period (2005–2012), 5456 women had an UADS performed at or after the 26th week of their pregnancy. Overall, 2712 (49.7%) women were excluded. Women were excluded either because of an IUGR fetus (*n* = 156), multiple gestation (*n* = 99), fetal congenital anomaly (*n* = 758), both IUGR and congenital anomaly (*n* = 106), or IUFD (*n* = 2). Women with missing delivery outcomes (*n* = 1436) or missing study variables (*n* = 155) were not included in our final analysis (Table 1). The final cohort included 2744 singleton pregnant women with normally grown fetuses.

**Table 1:** Maternal and obstetric characteristics according to umbilical artery Doppler studies

Characteristics	Total *N* = 2744	No (%) in group		*P* value
		Normal UADS *N* = 2646	Abnormal UADS *N* = 98	
Maternal age, mean (standard deviation)	28.8 (6.6)	28.7 (6.6)	29.7 (6.7)	NS *
Race (%)				< 0.05 §
White	904	881 (33.3)	23 (23.5)	
African-American	1422	1356 (51.3)	66 (67.4)	
Hispanic	70	69 (2.6)	1 (1.0)	
Asian	231	226 (8.5)	5 (5.1)	
Other	117	114 (4.3)	3 (.3.1)	
17 missing				
Parity (%)				NS §
Nulliparous	1246	1202 (45.4)	44 (44.9)	
Primiparas	839	813 (30.7)	26 (26.5)	
Parity = 2	344	327 (12.4)	17 (17.4)	
Parity = 3	181	174 (6.6)	7 (7.1)	
Parity > 3	134	130 (4.9)	4 (4.1)	
Hypertension (%)				< 0.001 §
None	2304	2234 (84.4)	70 (71.4)	
Chronic	186	175 (6.6)	11 (11.2)	
Gestational	103	100 (3.8)	3 (3.1)	
Superimposed	42	37 (1.4)	5 (5.1)	
Preeclampsia	109	100 (3.8)	9 (9.2)	
Diabetes (%)				NS §
None	2321	2244 (84.8)	77 (78.6)	
Chronic	138	129 (4.9)	9 (9.2)	
Gestational	285	273 (10.3)	12 (12.2)	
Gestational age at delivery, median (IQR)	39.1 (38–40)	39.1 (38.1–40)	38.6 (37.3–39.7)	< 0.001 ¶
Fetal gender (%)				NS §
Male	1409	1361 (51.4)	48 (49)	
Female	1335	1285 (48.6)	50 (51)	
Low-lying placenta (%)				NS §
No	2649	2556 (96.6)	93 (94.9)	
Yes	95	90 (3.4)	5 (5.1)	
Placenta previa (%)				< 0.05 §
No	2730	2634 (99.6)	96 (98.0)	
Yes	14	12 (0.4)	2 (2.0)	
PPROM (%)				NS §
No	2669	2574 (97.3)	95 (96.9)	
Yes	75	72 (2.7)	3 (3.1)	
Smoker (%)				NS §
No	2578	2489 (94.1)	89 (90.8)	
Yes	166	157 (5.9)	9 (9.2)	
Delivery mode (%)				NS §
Vaginal Delivery	1501	1454 (55.0)	47 (48.0)	
Operative	251	241 (9.1)	10 (10.2)	
Primary CS	601	575 (21.7)	26 (26.5)	
Repeated CS	391	376 (14.2)	15 (15.3)	
Pre-pregnancy BMI (%)				NS §
Underweight	135	130 (4.9)	5 (5.1)	
Normal	1193	1151 (43.5)	42 (42.9)	
Overweight	547	533 (20.1)	14 (14.3)	
Obese	869	832 (31.4)	37 (37.8)	
138 missing				

*UADS* Umbilical artery Doppler study, *IQR* Interquartile range, *NS* Not significant

* Two-sample T-test used; § Chi-square test used; ¶ Mann-Whitney U test used

### Descriptive data

Of the 2744 women, 98 (3.6%) had an abnormal UADS. Table 1 shows the characteristics of our cohort based on UADS. On univariate analysis, the groups were similar except for racial-ethnic background, hypertension status, placenta previa, and gestational age at delivery. Women who had an abnormal UADS were more likely to be African Americans, more likely to be diagnosed with hypertensive diseases and placenta previa, and more likely to deliver at an earlier gestational age (*p* <  0.05) (Table 1).

Collinearity between the variables was low with a mean variance inflation factor of 1.78. There was a strong positive correlation between each pairwise combination of S/D and PI (Spearman’s rho = 0.94), S/D and RI (Spearman’s rho = 0.97), PI and RI (Spearman’s rho = 0.92), suggesting collinearity between these parameters.

### Small for gestational age

The overall SGA rate was 13.8% (379/2744) among our cohort. SGA neonates had a higher frequency of an abnormal UADS (6.9%) than non-SGA neonates (3.0%) (*p* <  0.001) (Table 2). This difference was observed regardless of how many abnormal Doppler parameters were observed (Table 2). Only one SGA neonate had absent end-diastolic flow and none of our cohort had reversed end-diastolic flow.

**Table 2:** Abnormal umbilical artery Doppler studies in small for gestational age neonates

	Total *N* = 2744	Number (%) in group		*P* value
		SGA *N* = 379	Non-SGA *N* = 2365	
Abnormal UADS (%)	98 (3.6)	26 (6.9)	72 (3.0)	< 0.001
One abnormal parameter (%)	38 (1.4)	8 (2.1)	30 (1.3)	< 0.001
Two abnormal parameters (%)	16 (.6)	4 (1.1)	12 (.5)	NS
Three abnormal parameters (%)	44 (1.6)	14 (3.7)	30 (1.3)	< 0.001
Abnormal S/D (%)	60 (2.2)	19 (5)	41 (1.7)	< 0.001
Abnormal PI (%)	78 (2.8)	21 (5.5)	57 (2.4)	< 0.001
Abnormal RI (%)	65 (2.4)	18 (4.8)	47 (2.0)	< 0.001

Chi square test was used

*UADS* Umbilical artery Doppler study, *S/D* Systolic-to-diastolic ratio, *PI* Pulsatility index, *RI* Resistance index, *SGA* Small for gestational age, *NS* Not significant

Women with an abnormal UADS were twice as likely to have an SGA neonate as women with a normal UADS (OR 2.35; 95% CI 1.48–3.72; *p* <  0.001). After adjusting for maternal age at delivery, racial-ethnic background, parity, smoking status, pre-pregnancy BMI, and fetal gender, the association between SGA and abnormal UADS was statistically significant (aOR 2.22; 95% CI: 1.38–3.58; *p* <  0.05).

In comparing different indices, out of 19 women who had an elevated S/D with an SGA neonate, 79% (15/19) had an elevated PI, while 89.5% (17/19) had an elevated RI. Abnormalities in S/D, PI, or RI were each statistically significantly associated with SGA neonates (*p* <  0.05) (Table 3).

**Table 3:** Logistic regression of abnormal umbilical artery Doppler studies and small for gestational age

	Unadjusted odds ratio (95% CI)	*P* value	Adjusted odds ratio (95% CI)^a^	*P* value
Abnormal UADS	2.35 (1.48–3.72)	< 0.001	2.22 (1.38–3.58)	< 0.05
One abnormal parameter	1.68 (.76–3.69)	NS	1.47 (0.66–3.30)	NS
Two abnormal parameters	2.09 (.67–6.52)	NS	2.02 (0.63–6.50)	NS
Three abnormal parameters	2.99 (1.57–5.68)	< 0.05	3.01 (1.55–5.86)	< 0.05
Abnormal S/D	2.99 (1.72–5.21)	< 0.001	2.99 (1.68–5.31)	< 0.001
Abnormal PI	2.38 (1.42–3.97)	< 0.05	2.22 (1.31–3.77)	< 0.05
Abnormal RI	2.46 (1.41–4.28)	< 0.05	2.46 (1.39–4.36)	< 0.05

*UADS* Umbilical artery Doppler study, *S/D* Systolic to diastolic ratio, *PI* Pulsatility index, *RI* Resistance index, *NS* Not significant

^a^Adjusted for maternal age at delivery, racial-ethnic background, parity, pre-pregnancy BMI, smoking, and fetal gender

### Other perinatal outcomes

The rates of NICU admission and low 5-min Apgar score among the cohort were 12.4% (340/2744) and 2.3% (62/2744), respectively. Table 4 shows the outcomes of NICU admission and low Apgar scores according to UADS results. NICU admission and low Apgar scores were more common among neonates with an abnormal UADS than neonates with a normal UADS. NICU admission was statistically significantly different between UADS groups, whereas low Apgar scores were not (Table 4). According to simple logistic regression, abnormal Doppler was statistically significantly associated with NICU admission (OR 2.39; 95% CI 1.49–3.85; *p* <  0.001). After we controlled for potential confounding factors, gestational age at delivery and birth weight, the adjusted odds ratio for NICU admission was 1.84 (95% CI 1.06–3.21; *p* <  0.05) (Table 5). In examining the association between low Apgar score and abnormal Doppler, there was no statistically significant association before or after adjusting for confounders (Table 5).

**Table 4:** Abnormal umbilical artery Doppler studies and adverse perinatal outcomes

Outcome	Total *N* = 2744	No (%) in group		*P* value
		Normal UADS *N* = 2646	Abnormal UADS *N* = 98	
SGA (%)	379 (13.8)	353 (13.3)	26 (26.5)	< 0.001
NICU admission (%)	340 (12.4)	316 (11.9)	24 (24.5)	< 0.001
Low Apgar score (%)	62 (2.3)	58 (2.2)	4 (4.1)	NS

Chi square test was used

*UADS* Umbilical artery Doppler study, *SGA* Small for gestational age, *NICU* Neonatal intensive care unit, *NS* Not significant

**Table 5:** Logistic regression of abnormal umbilical artery Doppler studies and other adverse perinatal outcomes

Outcome	Unadjusted odds ratio (95% CI)	*P* value	Adjusted odds ratio (95% CI)	*P* value
NICU admission^a^	2.39 (1.49–3.85)	< 0.001	1.84 (1.06–3.21)	< 0.05
Low Apgar score^b^	1.90 (0.68–5.34)	NS	1.39 (0.47–4.07)	NS

NICU, neonatal intensive care unit; NS, not significant

^a^Adjusted for gestational age at delivery and birth weight

^b^Adjusted for gestational age at delivery

### Subgroup analysis

We examined the associations between abnormal UADS, NICU admission, and low Apgar score among SGA neonates. In this subgroup, the rates of admission to the NICU and low Apgar score were 10.6% (40/379) and 2.1% (8/379), respectively. Abnormal UADS was statistically significantly associated with NICU admission (OR 4.46; 95% CI 1.80–11.06; *p* <  0.05), and this association persisted after adjusting for gestational age at delivery (aOR 2.91; 95% CI 1.06–7.97, *p* <  0.05). None of the eight SGA neonates who had low Apgar scores had an abnormal UADS.

## Discussion

### Main findings of the study

Our data demonstrate that abnormal UADS in fetuses that appear normally grown by ultrasound is statistically significantly associated with SGA and NICU admission. We also found a strong association between abnormal UADS and NICU admission in the subgroup of neonates with SGA.

### Comparison with findings from previous studies

To our knowledge, this is the largest study to date investigating UADS in normally grown fetuses. A prospective cohort study by Bolz et al. investigated the use of UADS in predicting SGA, where elevated PI was associated with higher rates of SGA neonates [14]. In that study, 30% of women with an elevated PI had an SGA neonate, similar to the percentage seen in our population. In contrast to our findings, Bolz et al. detected only one neonate with a prenatally elevated PI who was admitted to the NICU and did not detect any neonates with an elevated PI who had a low 5-min Apgar score [14]. A multicenter prospective study by Goffinet et al. found that normally grown fetuses with prenatally elevated RI had twice the odds of developing [15]. About 10% of women with an elevated RI had their neonate transferred to the NICU but associations were not investigated. In contrast to our findings, none of the women with an abnormal Doppler delivered a neonate with a low 5-min Apgar score [15]. Filmar et al. found that an elevated S/D ratio in normally grown fetuses was associated with increased risks of SGA neonates and NICU admission, consistent with our findings [16]. However, our study included a broader definition of abnormal UADS (elevated S/D, PI, or RI, individually or cumulatively) than Filmar’s study, which only included elevated S/D ratio [16]. A retrospective cohort study by Khalil et al. found that elevated PI was significantly associated with NICU admission in both normally grown fetuses and those with IUGR [17]. An additional population-based, prospective study in the Netherlands reported elevated PI associated with SGA; however, they did not specify the exclusion of IUGR fetuses [20].

Our analysis of the subgroup of SGA neonate showed a strong association between abnormal UADS and NICU admission. In contrast, Khalil et al. reported that elevated PI was not associated with NICU admission in SGA neonates [17]. Dicke et al. reported that prematurity is an important predictor of NICU admission in SGA neonates with IUGR rather than an abnormal S/D and PI or abnormal S/D alone. However, this study did not investigate SGA neonates with normal in utero growth [21].

### Strengths and limitations of the study

The strengths of our study include the use of a large cohort of women and appropriate comparison groups. All of the subjects had their ultrasounds and delivered their babies at our institution, which minimizes selection bias and the heterogeneity of ultrasonographer and physician practice. Electronic medical records were used to extract the data, which minimizes recall bias. Efforts were made to reduce missing data; when there were missing ultrasound measures, original ultrasound images were obtained. Women in our study may have had more than one UADS performed during their pregnancy, but only the last one before delivery was included. This was appropriate to account for women who had improved UADS before delivery.

This study does have some limitations, including its retrospective design and lack of outcome data for subjects who did not deliver at our institution. Smoking and pre-pregnancy BMI were adjusted for in our final model; however, the duration and amount of smoking were unknown, and weight gain was not consistently documented throughout pregnancy. Some potential confounders, such as socioeconomic status, previous SGA, and other medical conditions, were not considered due to lack of data. Finally, this study may not be representative of other clinical settings.

## Conclusions

In summary, our data show a significant association between abnormal UADS and SGA among fetuses thought to be appropriately grown on the most recent ultrasound prior to their delivery. While we are not proposing that UADS is a suitable screening tool for SGA, if an abnormal UADS is detected on ultrasound, clinicians should be aware that their patient is at greater risk to have a neonate with an SGA birth weight or who requires NICU care.
